# Supplementary material for: An implantable soft robotic ventilator augments inspiration in a pig model of respiratory insufficiency
Source: Nat Biomed Eng. 2022 Dec 12;7(2):110–23. doi: 10.1038/s41551-022-00971-6 (PMC9991903; doi:10.1038/s41551-022-00971-6)
Supplement: Supplementary file 1 — Supplementary figures, notes, methods, tables and references. [file 41551_2022_971_MOESM1_ESM.pdf]

nature biomedical engineering

Article

https://doi.org/10.1038/s41551-022-00971-6

An implantable soft robotic ventilator augments inspiration in a pig model of respiratory insufficiency

In the format provided by the authors and unedited

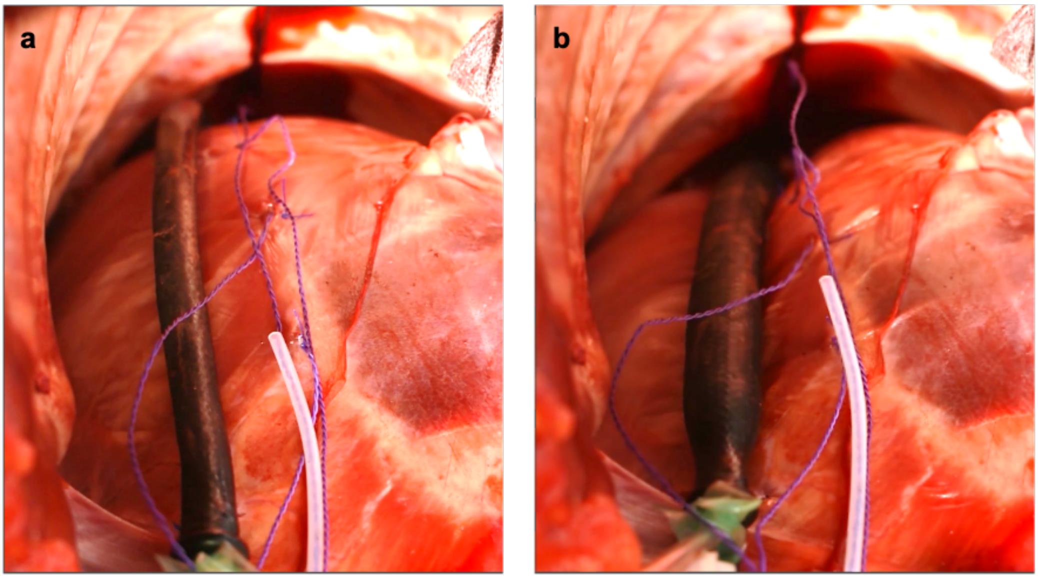

Supplementary Fig. 1 I Visualization of an actuator in situ. Supradiaphragmatic images of a single McKibben actuator placed *in situ* on the right hemidiaphragm in an **(a)** unpressurized and **(b)** pressurized (20 psi) state. The top of the images is the dorsal side. The bottom of the images is the ventral side. The anterior attachment point of the actuator is attached to the peristernal area. The posterior attachment point of the actuator is passed through the last intercostal space and sutured to the skin.

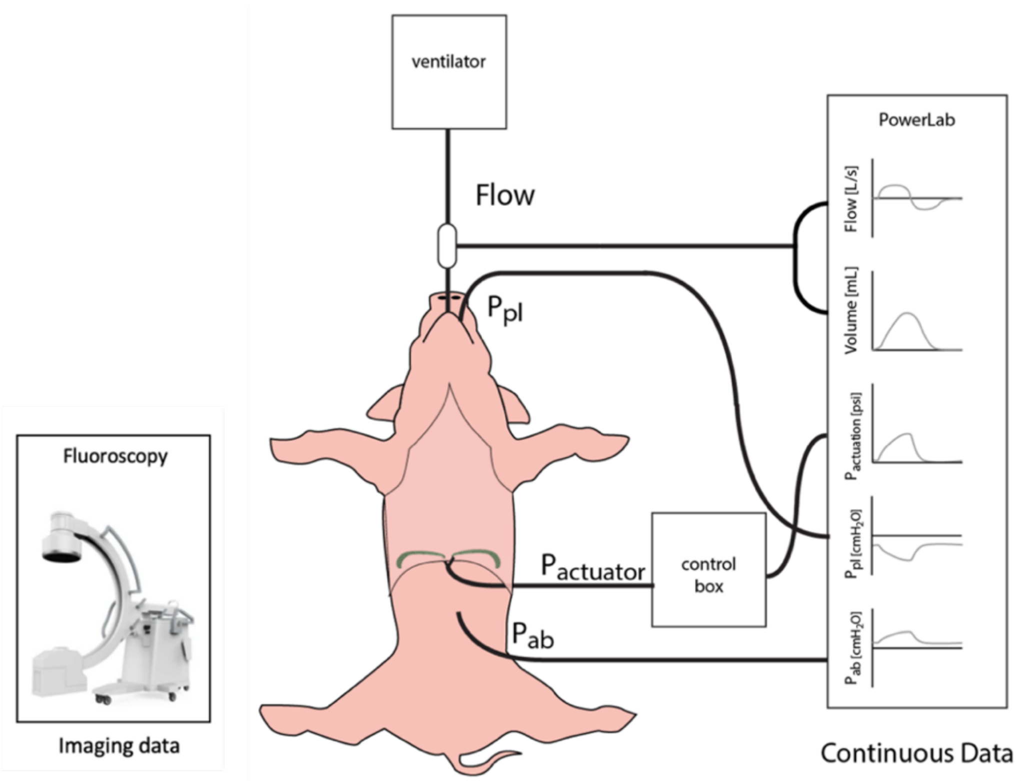

Supplementary Fig. 2 I Bioinstrumentation set up. Schematic of the bioinstrumentation setup to collect different types of physiological data. For the continuous data that was acquired via the PowerLab data acquisition system, idealized waveforms of one period of each signal are depicted.

Supplementary Notes

Controlling PAM actuator performance via pressurization

Actuator performance was characterized both *in vitro* and *in vivo* as described in the Methods in the main manuscript and the Supplemental Methods presented below.

Different input shapes explore the effect of rate of pressurization (Extended Data Fig. 1a-c). The fidelity to these idealized waveforms is limited by the control resolution of the electropneumatic regulators, and ultimately result in the output pressurization curves of Extended Data Fig. 1d-f. These actuation pressure curves ultimately govern the mechanical performance of the actuators. The actuators are characterized *in vitro* via tensile and flexural testing, as described in the Supplemental methods. The tensile force (Extended Data Fig. 1g-i) represents the contractile force applied to the points of attachment on the ribs, and the flexural force (Extended Data Fig. 1j-l) represents the force perpendicular to the actuator towards the diaphragm.

Different actuation pressure waveforms result in different displacements, (seen in the M-mode ultrasound in Extended Data Fig. 1m-o and quantified in Extended Data Fig. 1p), tidal volumes (Extended Data Fig. 1q) and different respiratory mechanics (Extended Data Fig. 1r). Notably, the square wave pressurization is distinct from the behavior of the curved wave and triangle wave, especially with regards to the average diaphragm displacement and the Campbell diagram. We note that the square wave achieves similar tidal volumes to the other waveforms while drawing more negative pleural pressures. The slope of the Campbell diagram, taken at the two points in the loop where $\frac{dV}{dP} = 0$, can be viewed as a representation of compliance of the system. A negative pleural pressure drives flow via the gradient from atmospheric pressure at the airway opening to the negative alveolar pressure, so we evaluate the absolute value of compliance. The slope generated by the square wave (12.1 mL/cmH_20) is considerably lower (i.e., the system is stiffer) than that of the curved (15.9 mL/cmH_20) and triangle wave (15.1 mL/cmH_20), which both have slopes that more closely resemble those that of spontaneous respiration (25.6 mL/cmH_20). These values are overall relatively stiff and are likely due to the low lung volumes for this subject. Qualitatively, we observe that the square wave results in “sharper” breaths that pull on the chest wall more aggressively compared to the gentler inflation of the curved and triangle wave, matching the much higher tensile forces generated by the square wave from *in vitro* testing.

The curved and triangular pressurization input are similar in their pressure-volume (PV) loops; however, the curved input achieves higher tidal volumes with marginally smaller levels of diaphragm displacement (Extended Data Fig. 1p-r), which could be attributed to the shorter time in which the actuator operates at a high pressure filled state which may not provide enough time for lung filling. Overall, the curved waveform used in the majority of the study (Fig. 2-7 in the main text) represents a pressurization scheme that aims to combine the benefits of the square wave and triangular wave, generating the best tidal volumes and biomimetic PV loops.

PAM performance can also be tuned via depth of pressurization by scaling the input curved waveform shown in Extended Data Fig. 2a to different peak pressures (5, 10, 15, and 20 psi), the resulting actuator pressure waveforms are shown in Extended Data Fig. 2a-d. The relationship between pressurization and forces generated is linear (Extended Data Fig. 2e,f) which corroborates previous McKibben characterization work[24]. We characterize the response of one subject to the varying degrees of pressurization. The degree of pressurization has a positive, but nonlinear effect on the amount of diaphragm displacement generated (Extended Data Fig. 2g-k). In this subject, we demonstrate tunability of the degree of augmentation via changes in pressurization, with the greatest range of responsiveness being between 0 and 10 psi. Additional increases taper off between 10 and 20 psi, which matches the understanding of how McKibben actuators operate, as they first expand and fill to their maximum volume, achieving maximum contraction, and beyond that they increase force generation[24]. In terms of respiratory mechanics, the degree of pressurization does not have a large effect on the change in pleural pressure (Extended Data Fig. 2m) unlike the different waveform shapes in Extended Data Fig. 2p.

Notably, inter-animal variability is undeniably a factor contributing to overall performance, as evident in the varied responsiveness to the device seen in Fig. 3. Even in a case of low augmentation, we saw a nonlinear but tunable response to different levels of pressurization. The absolute degrees of augmentation shown in Extended Data Fig. 2k-m will obviously not hold across different animals, but we expect that the relative effect of tuning pressure should.

Complete arterial blood gas results

Supplementary Table 1 depicts ABGs taken during the respiratory challenges depicted in Fig. 5 with (a) independent actuation, (b) synchronized actuation, and (c) synchronized actuation after 2 minutes of unsupported ventilation. During (a), we observe a progressive blood acidification, a consequence of increasing P_aCO_2 (respiratory acidosis). In parallel, HCO_3 increases, and is an adaptative mechanism of the organism to correct pH value. However, this compensatory mechanism only partially helps controlling pH. During synchronized actuation (b), P_aCO_2 remains constant around the upper level of the normal range. We see a progressive acidification of the blood, whose main primary cause is a decrease in HCO_3 (metabolic acidosis). For respiratory challenge (c), ABGs shows a clear respiratory acidosis (elevated P_aCO_2) with partial compensatory mechanism (increase in HCO_3) during the unsupported ventilation phase. While actuating, P_aCO_2 decreases, as well as HCO_3, resulting in a controlled pH. At the end of the trial, P_aCO_2 slightly increases with a corresponding HCO_3 compensation. P_aO_2 and O_2 saturation (S_aO_2) remained in the normal range across all experiments.

a

**Time [s]**	**0**	**370**	**618**
**pH**	7.449	7.307	7.294
**pCO2 [mmHg]**	37.8	89.2	68
**HCO3 [mmol/l]**	26.4	45	33.3
**pO2 [mmHg]**	313.4	228.2	189
**sO2 [%]**	99	100	99

b

**Time [s]**	**0**	**262**	**626**
**pH**	7.408	7.332	7.285
**pCO2 [mmHg]**	47.4	44.9	47.2
**HCO3 [mmol/l]**	30.2	24	22.6
**pO2 [mmHg]**	246	217.9	158.2
**sO2 [%]**	99	100	100

c

**Time [s]**	**0**	**124**	**433**	**732**	**1010**
**pH**	7.471	7.337	7.359	7.419	7.389
**pCO2 [mmHg]**	35.6	75.1	63.2	33.6	54.7
**HCO3 [mmol/l]**	26.2	40.6	36	21.9	33.4
**pO2 [mmHg]**	256.6	233.1	292.4	232.7	272.2
**sO2 [%]**	99	99	100	100	99

Supplementary Table 1 | Full arterial blood gas results. Arterial blood gas results for the respiratory challenges reported in Fig. 5. Here, Table 1 a,b,c corresponds with Fig. 5a,b,c respectively.

Further data on the timing alignment analysis

As discussed in the main manuscript in the section entitled "Factors in optimizing synchronization", there are many different alignment factors that can be analyzed (Supplementary Fig. 3). One additional metric of interest is the relationship between the start of actuation (P_0) and the start of inspiration (V_0). Because the triggering system presented here relies on the beginning of inspiration for triggering, this is a metric that we hypothesized would be influential. We find no linear relationship between (P_0-V_0) and the tidal volume or the peak inspiratory flow (Supplementary Fig. 4). Although we see a clustering effect in Supplementary Fig. 4a,c, within the clusters, there are no linear relationships. The clusters themselves can be understood as two categories of actuation. Actuation that occurred early (likely falsely triggered) with a negative P_0-V_0 value and actuation that occurred in an appropriately synchronized fashion. With this distribution of behavior within one respiratory challenge, the data actually resembles the data from comparing independent and synchronized trials (Fig. 5e,f). If we solely look at the data with a positive P_0-V_0 for all of Fig. 4, we find these clusters to both lack any linear relationship internally. This contrasts with the linear relationships found relating P_0 and V_{pk}.

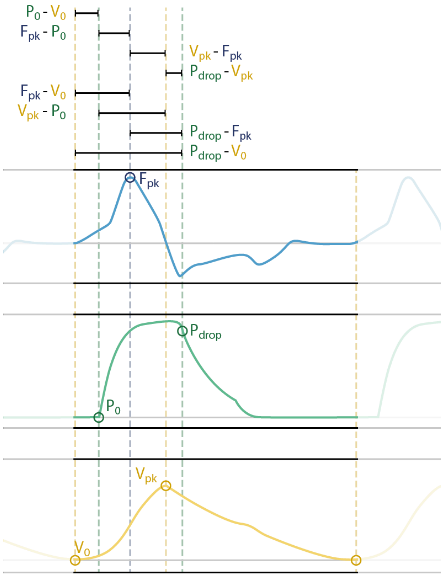

Supplementary Fig. 3 I Alignment metrics. Schematic depicting many of the alignment metrics derived from the flow, pressure, and volume waveforms that can be used for synchronization. P_0: start of actuation, V_0: start of inspiration, V_{pk}: start of expiration, F_{pk}: peak inspiratory flow, P_{drop}: start of actuator deflation.

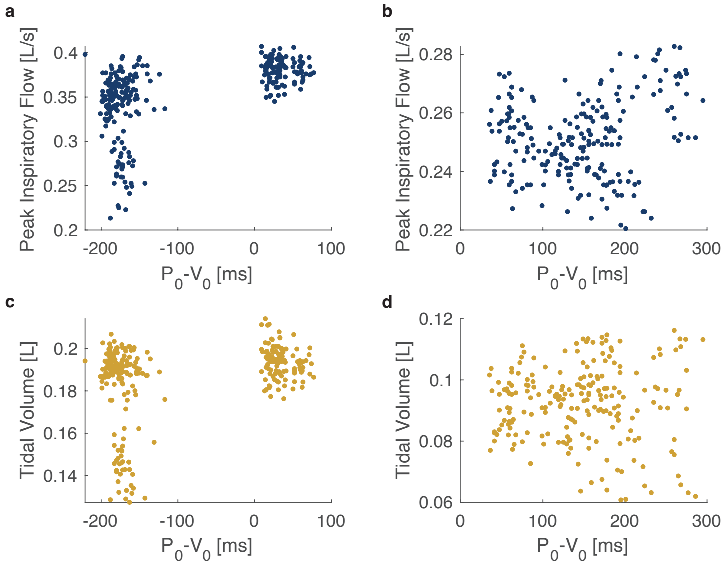

Supplementary Fig. 4. Effect of the alignment of P_0-V_0 on flow and volume. a,b, A scatter plot of peak inspiratory volume as it relates to the time between P_0 and V_0 for one respiratory challenge with an (**a**) intact

phrenic nerve (278 breath) and with a (**b**) severed phrenic nerve (215 breaths). **c,d**, A scatter plot of tidal volumes as it relates to the time between P_0 and V_0 for one respiratory challenge with an (**c**) intact phrenic nerve (278 breath) and with a (**d**) severed phrenic nerve (215 breaths). All data is taken from the same subject (one biological replicate). Each dot represents one technical replicate (one breath). P_0: start of actuation, V_0: start of inspiration.

Considerations for PAM materials selection

Due to the acute nature of the study, we did not test the biocompatibility of the actuators. We used commercially available materials for ease of prototyping. In our design process, we did select materials with consideration of their future biocompatibility. PET, which makes up the mesh of our actuator, was chosen for its mechanical strength and chemical stability and has been used as a biocompatible material for vascular prostheses under the trade name Dacron[50,51]. Polyurethanes have been used in many biomedical applications for their biocompatibility and mechanical stability.[20,52,53] The thermoplastic elastomer of the balloon has been used in research studies creating patient specific valves[20] and neurosurgical tools[57]. Overall, the mechanical nature of our device is agnostic to specific material chemistry beyond the mechanical properties of different materials. Therefore, these components can easily be substituted for regulatory-approved biomaterials.

Supplementary Methods

McKibben Pneumatic Artificial Muscle (PAM) Manufacturing

The McKibben actuators used in this study consist of a thermoplastic elastomer bladder (Stretchlon 200, FibreGlast Developments Corp., Brookville, OH, USA), a thermoplastic polyurethane tubing (1/8" Tubing, 5648K226, McMaster-Carr, Inc, Elmhurst, IL, USA) and a poly(ethylene terephthalate) (PET) expandable braided mesh (PTO0.25BK, TechFlex, Inc., Sparta, NJ, USA). These actuators were sized to fit our 30-40 kg Yorkshire Swine.

First, the internal bladder was fabricated and coupled with the airline. The elastomeric bladder was formed by heat-sealing. Two layers of thermoplastic elastomer were aligned on top of a 3D-printed mold (Objet 20 Pro Stratysys, Ltd., Eden Prairie, MN, USA) and heat sealed with a heat press (Fancierstudio, Hayward, CA, USA) at 300°F for 5 s. The 3d printed mold yielded a bladder with dimensions 20 cm by 5 cm with a negative space for the airline measuring 1/8 in wide to accommodate the outer diameter of the tubing. After heat sealing, the polyurethane tubing was inserted into the airline opening. This junction was sealed with a urethane adhesive (Ure-Bond II, Smooth-On, Inc, Macungie, PA, USA).

Next, the bladder was coupled with the expandable braided mesh (PTO0.25BK, TechFlex, Inc.). Mesh was cut to a relaxed length of 26 cm. The bladder was placed inside the tubular mesh. To constrain the two ends of the active contractile region of the actuator, Kevlar thread (McMaster-Carr, Inc, Elmhurst, IL, USA) was used to sew the mesh closed at the two endpoints of the actuator. The active contractile length of the relaxed mesh was set to 18 cm, the distance between the two Kevlar points of closure. At the end of this process, the two most terminal ends of the mesh were heated and fused together to prevent fraying of the non-active ends of the mesh, resulting in an actuator with a total relaxed length of 25cm.

After fabrication, actuators were subject to fatigue testing of >1000 cycles of pressurization to 20 psi as a quality control check to reveal manufacturing defects. During this testing, actuators were restrained on a custom 3-D printed scaffold—a modified flex fixture—mounted onto a stationary Instron testing setup (Supplementary Fig. 5) that secured the actuator in an initially curved position.

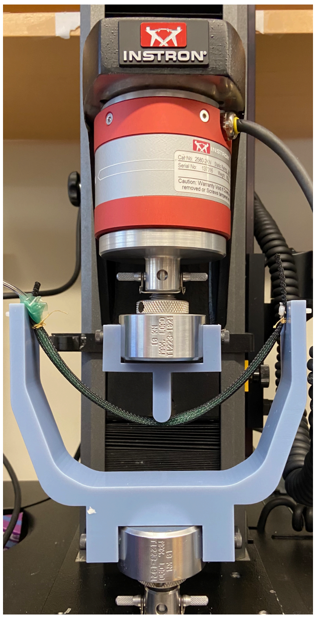

Supplementary Fig. 5 l Modified flex fixture set up. Image of the actuator fixed to a custom 3d-printed modified flex fixture setup used for mechanical characterization and fatigue testing.

McKibben PAM Mechanical Characterization Methods

The forces generated by the McKibben actuators were characterized by two experimental methods. One method used standard fixed length contractile (isometric) measurements. Each end of the actuator was fixed to standard Instron tensile grips with the actuator at its relaxed length. The actuator was pressurized to 20 psi and its contractile force was measured.

Additionally, the McKibben actuators were placed into a custom modified flexural fixture, described in Fig. S5. Upon actuation, the contraction of the actuator pushed the actuator body into the 3-D printed convex component, providing a resistance to actuator straightening. This was intended to measure the bending load and simulate a resistive load of the dome of the diaphragm, although this set up represented a higher resistance than the dynamic and compliant diaphragm.

Prior to use, actuators underwent fatigue testing in this in the modified flex fixture set up shown in Supplementary Fig. 5. Actuators that survived >1000 cycles of pressurization to 20 psi via the standard curved input waveform on the benchtop did not fail during the subsequent >1000 cycles of pressurization to 20 psi in the *in vivo* studies. The actuators that underwent the greatest total number of cycles withstood >3000 cycles and were still intact at the end of use.

PAM contraction and mechanical performance is determined by the controlled pressurization of the actuators. Preset pressurization waveforms are programmed into the microcontroller of the control system and given as an input to the electropneumatic regulators. We characterize the actuator behavior for different shapes and depths of pressurization.

Three different input shapes are shown in Extended Data Fig. 1a-c. All three waveforms represent a single breath with a timescale of 33 breaths per minute (1.82s per breath), an inspiratory time to expiratory time ratio (I:E) of 0.5, and a maximum pressurization of 20 psi. Extended Data Fig. 1a represents our curved waveform constructed to replicate native respiratory waveforms when used to drive respiration in our previously reported respiratory simulator[56]. Extended Data Fig. 1b represents the corresponding square wave to the above parameters. Extended Data Fig. 1c represents a triangle wave intended to mimic the rise and fall of the curved waveform.

To investigate the effect of depth of pressurization, the curved waveform of Extended Data Fig. 1a is scaled to different levels of pressurization (5, 10, 15, 20 psi).

References

57. Amadeo, T. *et al.* Soft Robotic Deployable Origami Actuators for Neurosurgical Brain Retraction. *Front. Robot. AI* **8**, 437 (2022).
