# Supplementary material for: An implantable soft robotic ventilator augments inspiration in a pig model of respiratory insufficiency
Source: Nat Biomed Eng. 2022 Dec 12;7(2):110–23. doi: 10.1038/s41551-022-00971-6 (PMC9991903; doi:10.1038/s41551-022-00971-6)
Supplement: Supplementary file 2 — Reporting Summary [file 41551_2022_971_MOESM2_ESM.pdf]

nature portfolio

Corresponding author(s): Ellen T. Roche

Last updated by author(s): Jun 23, 2022

Reporting Summary

Nature Portfolio wishes to improve the reproducibility of the work that we publish. This form provides structure for consistency and transparency in reporting. For further information on Nature Portfolio policies, see our Editorial Policies and the Editorial Policy Checklist.

Statistics

For all statistical analyses, confirm that the following items are present in the figure legend, table legend, main text, or Methods section.

n/a	Confirmed	
☐	☒	The exact sample size (*n*) for each experimental group/condition, given as a discrete number and unit of measurement
☐	☒	A statement on whether measurements were taken from distinct samples or whether the same sample was measured repeatedly
☐	☒	The statistical test(s) used AND whether they are one- or two-sided<br>*Only common tests should be described solely by name; describe more complex techniques in the Methods section.*
☒	☐	A description of all covariates tested
☒	☐	A description of any assumptions or corrections, such as tests of normality and adjustment for multiple comparisons
☐	☒	A full description of the statistical parameters including central tendency (e.g. means) or other basic estimates (e.g. regression coefficient) AND variation (e.g. standard deviation) or associated estimates of uncertainty (e.g. confidence intervals)
☐	☒	For null hypothesis testing, the test statistic (e.g. *F*, *t*, *r*) with confidence intervals, effect sizes, degrees of freedom and *P* value noted<br>*Give P values as exact values whenever suitable.*
☒	☐	For Bayesian analysis, information on the choice of priors and Markov chain Monte Carlo settings
☒	☐	For hierarchical and complex designs, identification of the appropriate level for tests and full reporting of outcomes
☒	☐	Estimates of effect sizes (e.g. Cohen's *d*, Pearson's *r*), indicating how they were calculated

Our web collection on statistics for biologists contains articles on many of the points above.

Software and code

Policy information about availability of computer code

Data collection	LabChart 8 software.
Data analysis	Data from LabChart 8 was exported to MATLAB R2021a. Data analysis was conducted via custom code written in MATLAB R2021a, to analyse parameters such as peak inspiratory flow and tidal volumes. The custom code is available at https://github.com/RocheLab/ImplantableVentilator.

For manuscripts utilizing custom algorithms or software that are central to the research but not yet described in published literature, software must be made available to editors and reviewers. We strongly encourage code deposition in a community repository (e.g. GitHub). See the Nature Portfolio guidelines for submitting code & software for further information.

Data

Policy information about availability of data

All manuscripts must include a data availability statement. This statement should provide the following information, where applicable:

- Accession codes, unique identifiers, or web links for publicly available datasets
- A description of any restrictions on data availability
- For clinical datasets or third party data, please ensure that the statement adheres to our policy

The main data supporting the findings of this study are available within the article and its Supplementary Information. Source data for the figures are provided with this paper. Additional data are available from the corresponding author on request.

Field-specific reporting

Please select the one below that is the best fit for your research. If you are not sure, read the appropriate sections before making your selection.

☒ Life sciences ☐ Behavioural & social sciences ☐ Ecological, evolutionary & environmental sciences

For a reference copy of the document with all sections, see nature.com/documents/nr-reporting-summary-flat.pdf

Life sciences study design

All studies must disclose on these points even when the disclosure is negative.

Sample size

We used a total of twelve swine during the development and testing of our system, and we present data from nine swine in the paper. For each subject, a series of respiratory challenges and conditions were tested. Different subsets of subjects were used for the experimental investigations reported; not all subjects were used in every experimental investigation. In this study, each breath provides a data point for the various analyses shown. A single respiratory challenge supplies a large pool of data.

Data exclusions

Data from nine swine are presented in this paper (six for quantitative data, and three for echocardiographic measurements). The data from the three swine not shown were not conducted under the same conditions, and therefore are not presented.

For Fig. 3, panels a and b are representative datasets. In this figure, the six swine presented are labeled A–F. Five of six swine (subjects A–E) are shown in Fig. 3c,d; subject F did not have data for the apnea condition at the start of the respiratory challenge and is therefore not shown.

Fig. 6 depicts the analysis of aligning the actuator synchronization to the underlying respiratory effort for two respiratory challenges within subject B.

Fig. 7 a–c omits subject C and F because the pressure-sensing instrumentation was nonfunctional for those two swine and the data for this parameter was not collected.

Replication

The trends shown in Fig. 6 are from one respiratory challenge within the animal. To ensure replication, this analysis was conducted on all data from this subject, and the reported trend replicates in a different respiratory challenge within the same animal.

For other subjects, the synchronization was actually overly consistent, and therefore lacked the variability in timing needed to visualize these trends. In order to replicate these results in another subject, a sweep of the synchronization/delay will generate the appropriate variability needed.

Randomization

The full set of respiratory challenges were conducted in each of 6 subjects, but in a semi-random order. The order of the independent and synchronous challenges was randomized, but the challenges conducted with a severed phrenic nerve necessarily came after all of the data with an intact phrenic nerve were collected. Echocardiography was performed in three swine.

Blinding

Individual subjects were not grouped and instead a variety of respiratory challenges were conducted within each subject.

Reporting for specific materials, systems and methods

We require information from authors about some types of materials, experimental systems and methods used in many studies. Here, indicate whether each material, system or method listed is relevant to your study. If you are not sure if a list item applies to your research, read the appropriate section before selecting a response.

Materials & experimental systems

n/a	Involved in the study	
☒	☐	Antibodies
☒	☐	Eukaryotic cell lines
☒	☐	Palaeontology and archaeology
☐	☒	Animals and other organisms
☒	☐	Human research participants
☒	☐	Clinical data
☒	☐	Dual use research of concern

Methods

n/a	Involved in the study	
☒	☐	ChIP-seq
☒	☐	Flow cytometry
☒	☐	MRI-based neuroimaging

Animals and other organisms

Policy information about studies involving animals; ARRIVE guidelines recommended for reporting animal research

Laboratory animals

Yorkshire swine, female, 30–40kg.

Wild animals

The study did not involve wild animals.

Field-collected samples	The study did not involve samples collected from the field.
Ethics oversight	All studies were conducted according to protocol #19-05-3907, approved by the Boston Children's Hospital (BCH) Institutional Animal Care and Use Committee (IACUC; policy and MIT protocol 0121-001-23).

Note that full information on the approval of the study protocol must also be provided in the manuscript.
